# Fatigue behavior of Ilizarov frame versus tibial interlocking nail in a comminuted tibial fracture model: a biomechanical study

**DOI:** 10.1186/1749-799X-1-16

**Published:** 2006-12-11

**Authors:** Erik Hasenboehler, Wade R Smith, Laurence Laudicina, Giby C Philips, Philip F Stahel, Steven J Morgan

**Affiliations:** 1 Department of Orthopaedic Surgery, Denver Health Medical Center, University of Colorado School of Medicine, 777 Bannock Street, Denver, CO 80204, USA; 2 Florida Sports Medicine Institute, 150 South Park Blvd., Suite 102, St. Augustine, FL 32086, USA

## Abstract

**Background:**

Treatment options for comminuted tibial shaft fractures include plating, intramedullary nailing, and external fixation. No biomechanical comparison between an interlocking tibia nail with external fixation by an Ilizarov frame has been reported to date. In the present study, we compared the fatigue behaviour of Ilizarov frames to interlocking intramedullary nails in a comminuted tibial fracture model under a combined loading of axial compression, bending and torsion. Our goal was to determine the biomechanical characteristics, stability and durability for each device over a clinically relevant three month testing period. The study hypothesis was that differences in the mechanical properties may account for differing clinical results and provide information applicable to clinical decision making for comminuted tibia shaft fractures.

**Methods:**

In this biomechanical study, 12 composite tibial bone models with a comminuted fracture and a 25 mm diaphyseal gap were investigated. Of these, six models were stabilized with a 180-mm four-ring Ilizarov frame, and six models were minimally reamed and stabilized with a 10 mm statically locked Russell-Taylor Delta™ tibial nail. After measuring the pre-fatigue axial compression bending and torsion stiffness, each model was loaded under a sinusoidal cyclic combined loading of axial compression (2.8/28 lbf; 12.46/124.6 N) and torque (1.7/17 lbf-in; 0.19/1.92 Nm) at a frequency of 3 Hz. The test was performed until failure (implant breakage or ≥ 5° angulations and/or 2 cm shortening) occurred or until 252,000 cycles were completed, which corresponds to approximately three months testing period.

**Results:**

In all 12 models, both the Ilizarov frame and the interlocking tibia nail were able to maintain fracture stability of the tibial defect and to complete the full 252,000 cycles during the entire study period of three months. A significantly higher stiffness to axial compression and torsion was demonstrated by the tibial interlocking nail model, while the Ilizarov frame provided a significantly increased range of axial micromotion.

**Conclusion:**

This is the first study, to our knowledge, which compares the biomechanical properties of an intramedullary nail to an external Ilizarov frame to cyclic axial loading and torsion in a comminuted tibia shaft fracture model. Prospective, randomized trials comparing Ilizarov frames and interlocked tibial nails are needed to clarify the clinical impact of these biomechanical findings.

## Background

Open fractures of the tibia with bone loss or extensive comminution can be treated by a variety of techniques [1-4]. A commonly well-accepted solution for tibia fractures is the interlocking tibial nail [5-9]. Rates of delayed unions and nonunions after intramedullary nailing range from 5% to 25% in the literature [3,5,10]. The concept of an external Ilizarov frame has also been recommended, but there are few reports specifically concerning the treatment of tibial shaft fracture management in the English language literature [11-13].

The present study on a biomechanical model was designed to investigate the fatigue behaviors of an interlocking tibial nail and the Ilizarov frame under a combined load of axial compression, bending and torsion. We believe that the understanding of the mechanical differences of both devices may provide new information applicable to clinical decision making in the treatment of comminuted tibial shaft fractures.

## Methods

**Figure 1:**
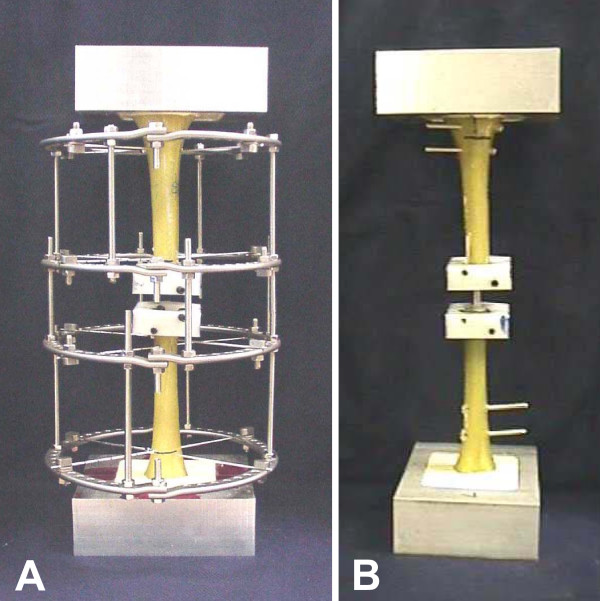
Overview of the biomechanical model systems used in this study: Ilizarov frame (**A**) and interlocking tibia nail (**B**). See text for details.

Twelve composite tibia bone models with a 25 mm diaphyseal gap were used for this biomechanical study to model a comminuted tibial fracture [14]. Six models were stabilized with an Ilizarov construct using eight 180-mm half rings and eight 1.8-mm olive wires tensioned to 130 kg. The other six models were minimally reamed and stabilized with a statically locked intramedullary nail (IMN) using a 10-mm Russell-Taylor Delta™ tibial nail and four 4.5-mm locking bolts [15]. Fig. 1 shows the models of the Ilizarov frame (**A**) and of the IMN construct (**B**).

Each construct was potted proximally and distally in a pair of loading fixtures, using Fast Cast^®^, and mounted on an 858 Bionix™ material-testing machine. To eliminate the potential for testing machine related data scatter, both the Ilizarov and IMN constructs were tested alternately on the two 858 Bionix™ systems. Custom-made loading fixtures were used to facilitate a clinically relevant combined loading of torque and axial compression bending with different proximal (23 mm) and distal (9 mm) offsets from the tibia's mechanical axis.

A linear variable differential transformer (LVDT) was mounted at the simulated fracture site to measure the fracture gap distance. The initial gap distance and pre-fatigue axial compression bending and torsion stiffness of both models were measured and documented prior to the start of the experiments. Axial deflection, torque and rotation were recorded by the LVDT (LabVIEW^® ^system). The stiffness was calculated from the slope of the load-deflection curve. A ramp compressive load at a rate of 0.2 in/min and a maximum of 178 N (40 lbf) was applied to observe bending stiffness in axial stress. For the torsion stiffness, a ramp torsion load at a rate of 5°/min and a maximum of 17 lbf-in (1.92 Nm) was applied.

Each model was subject to three consecutive cycle periods of 84,000 cycles, of which the last was used to determine the frames' bending and torsion stiffness in axial and torsion load. Thereafter, each model was mounted under a sinusoidal cyclic combined loading of axial compression of 2.8/28 lbf (12.46/124.6 N) and torque of 1.7/17 lbf-in (0.19/1.92 Nm) at a frequency of 3 Hz. Load was applied until either failure occurred, as defined by an implant breakage or ≥ 5° angulation and/or 2 cm shortening, or when the three cycle periods of 252,000 cycles were completed, which corresponds to a simulated clinical loading time of approximately 3 months. Every 84,000 cycles the test was interrupted to re-measure the stiffness and the gap distance under zero load. The applied loading stress which was estimated to be clinically relevant has previously been determined in a different biomechanical study using unilateral external fixators [16,17].

All the data were collected and analyzed by Lab View^® ^software and statistical analysis was performed by ANOVA with a *P*-value < 0.05 being considered statistically significant.

## Results and discussion

**Figure 2:**
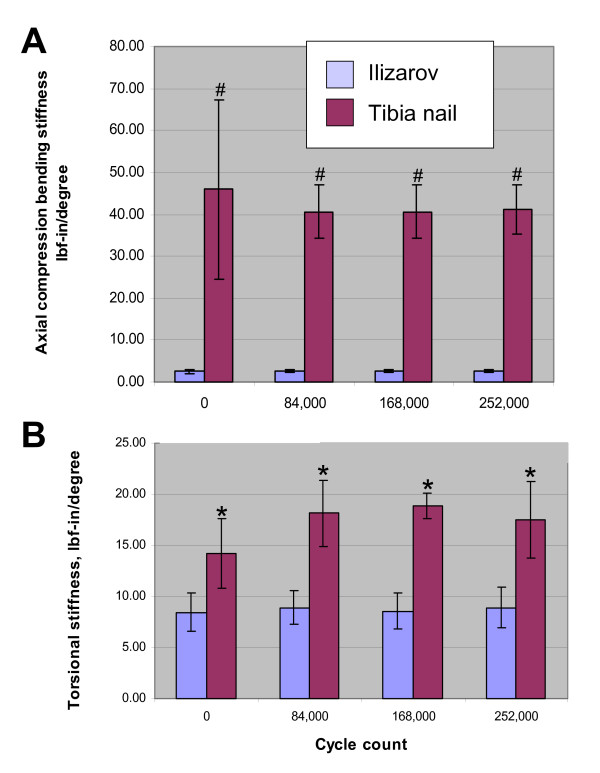
Comparison of axial compression bending stiffness (**A**) and torsion stiffness (**B**) of the Ilizarov frame compared to the tibial interlocking nail system over time/cycle counts. Data are shown as means ± SD of *n *= 6 biomechanical systems tested for each modality. *P < 0.05 and ^#^*P *< 0.01.

All our 12 model systems could successfully conclude the 252,000 cycles without any implant breakage or deformity equivalent to clinical complications, such as ≥ 5° angulation and/or ≥ 2 cm shortening. Neither the axial compression bending nor the torsion stiffness was shown to change statistically over time within the individual groups (Fig. 2, *P *> 0.05). Similarly, no significant difference of the gap distance change over time/cycles was observed within the individual groups (*P *> 0.05; data not shown). However, a significant reduction in axial compression bending stiffness (2.56 ± 0.34 *vs*. 42.22 ± 11.77 lbf-in/degree, mean ± SD, Ilizarov *vs*. IMN, Fig. 2A) and of torsion stiffness (8.71 ± 1.71 *vs*. 17.05 ± 3.46 lbf-in/degree, mean ± SD, Ilizarov *vs*. IMN, Fig. 2B) of the Ilizarov frame was detected at all cycle loads assessed, as compared to the IMN model. Furthermore, the Ilizarov frame model showed a statistically significant increase in maximum gap distance change, corresponding to increased micromotion, compared to the tibia nail (0.749 ± 0.010 mm *vs*. 0.009 ± 0.006 mm, mean ± SD, Ilizarov *vs*. IMN, *P *< 0.05).

This study was designed to assess the biomechanical properties of locked IMN and external Ilizarov frames in a comminuted tibia shaft fracture model. Several publications have previously analyzed the different biomechanical aspects of the Ilizarov frame fixators compared to unilateral or hybrid external fixators [20, 21, 22, 23, 26, 27, 28, 29, 30, 31, 32, 33]. This is the first report, to our knowledge, which describes the comparison of biomechanical properties of an Ilizarov frame versus an interlocking nail in a comminuted tibia fracture model. Interestingly, the amplitude of the change in fracture gap distance and the stiffness remained unaltered within the individual groups (Ilizarov and IMN) throughout the entire testing period, implicating that both constructs were able to maintain fracture stability. Likewise, neither model lead to a permanent deformity in terms of a malalignement. A composite tibia was chosen over a cadaveric model due to the more standardized features under different loading stresses [18]. The comminuted fracture model was selected for this study as severe tibial fractures present a clinical challenge and demonstrate a high rate of complications [3,10,13,19]. Intramedullary nails are well accepted for tibial shaft fractures, however, comminuted severe fractures still demonstrate nonunion rates of 5% to 25% [3,5,10]. In the international literature, Ilizarov external fixation is considered an indication for tibial fractures with comminution, significant bone loss, periarticular fractures or treatment for complications such as nonunion, malunion, infection or leg length discrepancy [11].

Our results indicate that both the Ilizarov frame and a statically locked intramedullary nail are able to maintain fracture stability over three months of normal clinical use in a comminuted tibial defect model. This model reflects a "worst case scenario", since under normal clinical conditions bone formation would typically occur enabling the bone to increasingly bear more load with time.

On other hand, since our model does not provide increasing stability at the fracture site due to callus formation over time, it must be considered a pure *"in vitro" *study. This model does not account for the potentially important biomechanical influence of the continuously changing stiffness due to the kinetics of fracture healing. However, as mentioned above, the composite tibia model offers the unique advantage of highly standardized biomechanical properties with regard to the reproducibility of different loading stresses, as opposed to the interspecimen variability in cadaveric or *"in vivo" *studies [18].

In this test design, the implants bore the full load throughout the duration of the test and healing callus did not influence biomechanics of fixation. Neither the intramedullary nail nor the Ilizarov frame failed in simulated weightbearing conditions over three months. This validates the immediate weightbearing concept of Illizarov and implies a similar potential for locked intramedullary tibial nails.

We utilized a simple four-ring, eight olive wire Ilizarov fixator construct for this study. Unilateral external fixators may demonstrate plastic or slip failure of frames during weightbearing with unstable fractures and frame fatigue may affect long-term interfragmentary stability [17]. The overall bending and torsion stiffness and shear rigidity of the Ilizarov external fixator are similar to those of conventional one-half pin fixators [20]. Ilizarov fixators demonstrate nonlinear mechanical properties in bending and nonlinear axial stiffness than do unilateral and bilateral external fixators. Wire size, tension, orientation as well as ring size and position contribute to overall frame rigidity and stability [21,22]. Increased Ilizarov stiffness can be achieved by bone preloading or compression, compressing rings together, increasing the number of wires and by using olive wires [21,22]. Wires crossed at 45° demonstrate greater torsional stiffness but less stiffness in axial compression and coupled axial compression significantly increases torsional stiffness [23]. In the present study, the stiffness of the IMN construct was significantly higher than that of the Ilizarov frame, however, the Ilizarov external fixator was able to provide good torsional resistance while allowing increased axial micromotion, a phenomenon which appears to stimulate callus formation [24,25].

## Conclusion

This biomechanical study on a comminuted tibia shaft fracture model demonstrates a significantly higher stiffness for axial compression and torsion by an interlocked tibia nail, as compared to an external Ilizarov frame. The Ilizarov construct, however, provided an increased axial micromotion. Prospective, randomized trials comparing Ilizarov frames and interlocked tibial nails are needed to clarify the clinical impact of these biomechanical findings.

## Competing interests

There are no financial interests by any of the authors regarding the present project.

## Authors' contributions

LL performed the biomechanical testing experiments and assisted with analysis of the data and writing of the manuscript. EH and GCP analyzed the data and wrote the final version of the manuscript. WRS, PFS, and SJM were responsible for conception and supervision of the study, planning of the experiments, and writing the manuscript.
